# Acute responses of inflammatory markers, glucose, and myoglobin in blood following neuromuscular electrical stimulation

**DOI:** 10.1007/s00421-026-06216-7

**Published:** 2026-04-02

**Authors:** Johanna Flodin, Parsia Amiri, Stefan M. Reitzner, Aisha S. Ahmed, Paul W. Ackermann

**Affiliations:** 1 Integrative Orthopaedic Laboratory, Department of Molecular Medicine and Surgery, Karolinska Institutet, Stockholm, Sweden; 2 Department of Trauma, Acute Surgery and Orthopaedics, Karolinska University Hospital, 171 76 Stockholm, Sweden; 3 Department of Physiology and Pharmacology, Karolinska Institutet, Stockholm, Sweden; 4 Department of Women’s and Children’s Health, Karolinska Institutet, Stockholm, Sweden; 5 Section of Orthopedics and Sports Medicine, Department of Molecular Medicine and Surgery, Karolinska Institutet, Stockholm, Sweden

**Keywords:** Electrical stimulation therapy, Inflammation, Muscle damage, Proteomics, Smart textiles, Glucose

## Abstract

**Purpose:**

Physical inactivity contributes to systemic inflammation and metabolic dysfunction. Neuromuscular electrical stimulation (NMES) offers an alternative to exercise for individuals unable to perform voluntary physical activity. This study investigated whether a single submaximal NMES session alters inflammation-related proteins, muscle-damage markers and glucose levels in healthy adults.

**Methods:**

Thirty-six healthy adults underwent a 2-hour NMES-session targeting the quadriceps, hamstring and gluteal muscles using wearable NMES-pants. Stimulation was delivered at individually tolerated intensities (visual analogue scale, (0–10), < 4). Venous blood samples were collected immediately before and after NMES. Inflammatory proteins and myoglobin were quantified using OLINK multiplex proteomic panel (92 proteins), and metabolic markers including glucose, lactate, pH and electrolytes were assessed using standard laboratory methods.

**Results:**

Of the 92 inflammatory proteins analyzed, 13 were nominally affected by NMES. After correction for multiple comparison, FMS-like tyrosine kinase 3 ligand (Flt3L) and fibroblast growth factor 19 (FGF19) remained significantly downregulated (Flt3L: 9.45 ± 0.12 to 9.11 ± 0.17, *p* < 0.001; FGF19; 9.43 ± 0.88 to 8.80 ± 0.84, *p* < 0.001; both normalized protein expression (NPX)). Myoglobin increased (7.70 ± 0.50 to 8.24 ± 0.83 NPX; *p* < 0.001), while blood glucose decreased modestly (5.35 ± 0.73 to 5.17 ± 0.36 mmol/L; *p* = 0.039). Lactate, pH, sodium and potassium remained unchanged.

**Conclusion:**

A single submaximal NMES-session elicited acute systemic responses characterized by downregulation of key inflammation-related proteins, mild myoglobin elevation and lower glucose levels – changes that mirror patterns observed after moderate exercise. These findings suggest that NMES at well-tolerated submaximal intensities can induce metabolic adaptations and may represent a safe, exercise-mimicking intervention to counteract inflammation and metabolic dysfunction in individuals with limited physical activity.

## Introduction

Physical inactivity is a major global health concern and the fourth leading cause of mortality worldwide (Guthold et al. 2018). It is strongly linked with non-communicable diseases such as cardiovascular disease, cancer, and type 2 diabetes, and is further exacerbated by increasingly sedentary lifestyles and aging populations (Guthold et al. 2018). In addition, physical inactivity promotes systemic low-grade inflammation which, particularly in post-surgical and immobilized patients, may delay recovery, impair wound healing, contribute to metabolic complications (Winkelman 2004) and increase the risk of deep vein thrombosis (Stone et al. 2017).

Neuromuscular electrical stimulation (NMES), which induces involuntary muscle contractions via externally applied electrical impulses, offers a promising alternative for individuals unable to engage in voluntary physical exercise (Hauger et al. 2018; Glaviano and Saliba 2016). Beyond preserving muscle activity, NMES has been associated with modulation of systemic inflammatory processes, with potential effects on tissue repair, vascular function and metabolic regulation (Fallah et al. 2022; Fouré and Gondin 2021). Technological advances, including wearable NMES-pants with integrated electrode arrays, have substantially improved usability, compliance and accessibility, enabling broader clinical and home-based applications (Maffiuletti et al. 2018; Gobbo et al. 2014; Euler et al. 2021; Flodin et al. 2022, 2023, 2024; Juthberg et al. 2023).

Previous work from our group has demonstrated that NMES enhances venous blood flow and modulates coagulation-related markers, thereby reproducing several hemodynamic effects typically observed during moderate physical exercise (Flodin et al. 2023, 2025; Sundström et al. 2023). However, the acute effects of submaximal NMES on circulating inflammation-related proteins, muscle damage markers and their interaction with metabolic pathways remain insufficiently understood.

Experimental and clinical studies suggest that NMES can induce acute cytokine responses, indicating that electrically evoked muscle contractions modulate inflammatory signaling in a manner comparable to exercise-induced immune responses (Truong et al. 2017; Steensberg et al. 2003). In the longer term, NMES has also demonstrated improvements in metabolic and neuromuscular function in both healthy and clinical populations (Griffin et al. 2009). Both NMES and voluntary exercise may induce low-grade muscle stress/damage, reflected by modest increases in circulating myoglobin (Ascensão et al. 2008; Del Coso et al. 2012; Flodin et al. 2024; Fouré and Gondin 2021; Yeung et al. 2002). In parallel, both modalities enhance glucose metabolism by activating contraction-dependent signaling pathways that promote glucose uptake and improve metabolic function (Steensberg et al. 2003; Alharbi et al. 2023; Hamada et al. 2003). Consistent with this, NMES has been shown to improve glycemic control in both healthy and clinical populations, supporting its potential as a therapeutic strategy for individuals with physical limitations and/or metabolic disorders (Alharbi et al. 2023; Sanchez et al. 2023).

We hypothesized that a single submaximal NMES session would induce measurable acute changes in circulating inflammation-related proteins, muscle-damage markers and glucose, with potential interactions affecting metabolic pathways relevant to physiological adaptation and recovery. To test this hypothesis, we investigated the effects of a single 2-hour NMES session on these biomarkers in peripheral blood from healthy adults. Wearable NMES-pants were used to stimulate the thigh and gluteal muscles at individually tolerated intensities (visual analogue scale, VAS < 4). By combining targeted proteomic profiling with established metabolic and muscle-damage markers (myoglobin, glucose, pH, lactate and electrolytes), this study builds on prior findings and provides new insights into the systemic biological effects of NMES, further clarifying its potential role as an exercise-mimicking intervention to mitigate the health risks associated with physical inactivity.

## Materials and methods

### Ethical approval

This study was performed in line with the principles of the Declaration of Helsinki. Ethical approval was obtained from the Regional Ethical Review Committees (Dnr: 2020–03374 and 2021 − 01439). All participants were provided with written and verbal information about the study design, procedures, and potential complications, and informed consent was obtained before participation in the study.

### Participants

A total of 36 healthy participants, aged between 18 and 55 years were included (Table 1). All participants reported their basic characteristics (age, sex, use of tobacco, physical activity level (PAS) (Frändin and Grimby 1994) in a questionnaire, and were measured in height and weight. Exclusion criteria were obesity (BMI > 30), pregnancy, skin ulcer, antithrombotic therapy, known vascular abnormalities, previous surgery in the deep vascular system of the leg, pacemaker, intracardiac defibrillator, advanced heart disease, kidney failure, neuromuscular and metabolic disease.

**Table 1:** Demographics and characteristics of the participants

Variable	Mean (standard deviation) (*n* = 36)
Sex, female, n (%)	16 (44.4)
Age, (years), M (SD)	31.9 (11.1)
Height, (cm), M (SD)	174.8 (10.4)
Weight, (kg), M (SD)	76.5 (20.0)
BMI, (kg/cm^2^), M (SD)	24.9 (5.89)
Smoker, n (%)	1 (2.8)
Physical activity level ^a^, M (SD)	4.22 (1.25)

*BMI* body mass index,* SD* standard deviation

^a^Frändin/grimby activity scale (1–6)

### Study design

**Fig. 1:**
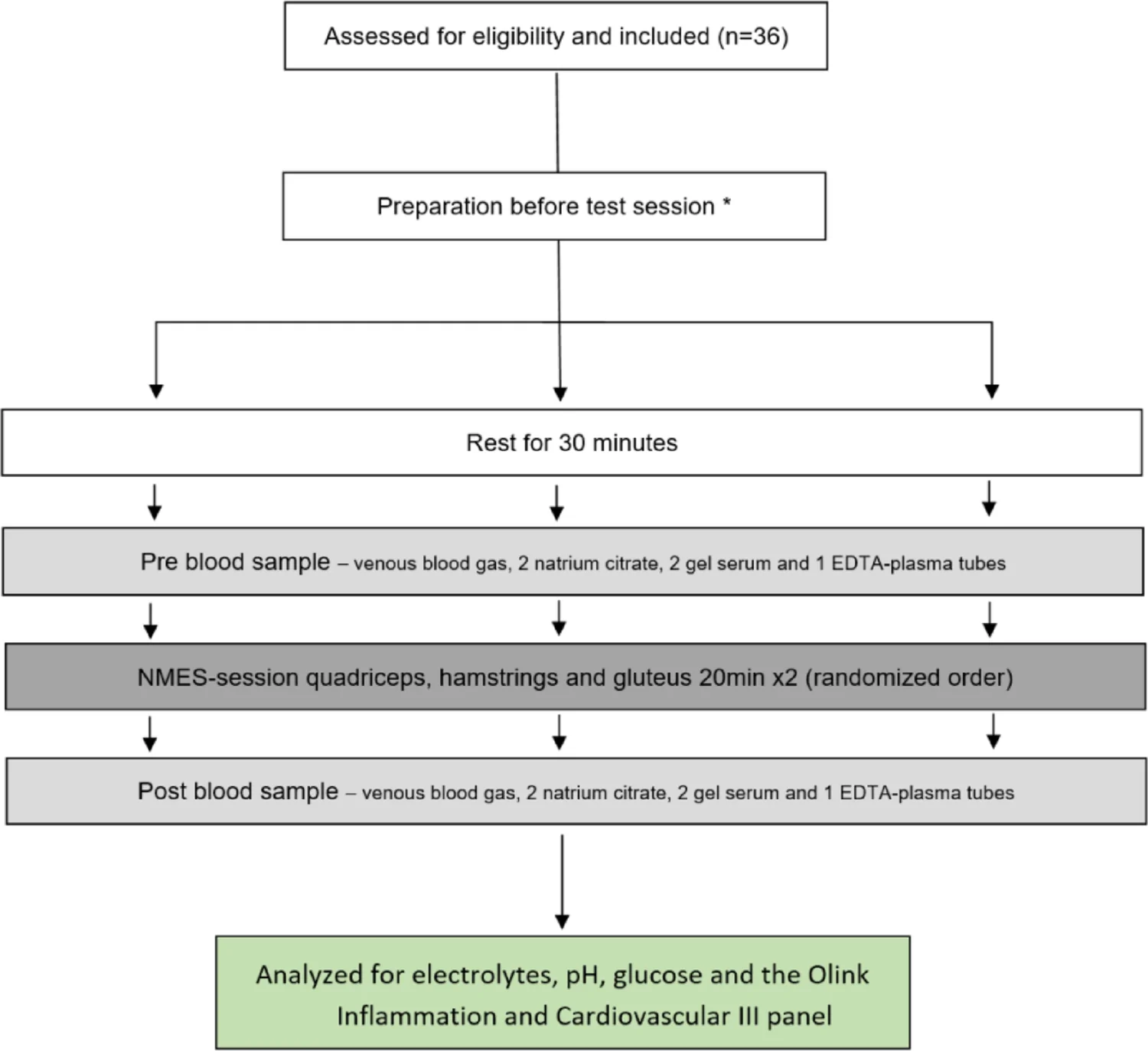
Consort flow chart of study design. Adapted from our previous study (flodin et al. 2025), but the green box describing the analyzed blood samples has been adjusted

In this study all participants performed one 2-hour NMES-session, with two timepoints for blood sample collection, e.g. before (pre) and after (post) the NMES-treatment. The study was performed in the same way as described in our previous study (Flodin et al. 2025). In brief, two 20-minutes NMES-sessions, session one (S1) and session two (S2), were performed on quadriceps (Q), hamstrings (H) and gluteus (G) muscle at an intensity-level providing a VAS just below 4 for each participant, which was defined as an acceptable level of pain based on previous studies (Myles et al. 2017). The study layout can be found in Fig. 1.

### Neuromuscular electrical stimulation

A constant-current NMES-device, Chattanooga Physio (DJO Nordic, Malmö, Sweden), utilizing a symmetrical, biphasic, square-form pulse, was employed for the NMES session. For NMES testing, all participants were supplied with a pair of NMES-pants containing sewn-in textile electrodes, as described in previous studies (Flodin et al. 2023). The participants put on the NMES-pants prior to the testing, and it was ensured that all electrodes made contact with the skin. Thereafter 10 ml conductive gel (Conductor transmission gel, Chattanooga, Eco-Med Pharmaceutical Inc., Canada) were applied on all of the electrodes. The participants were then placed in the same position as described in detail in our previous study (Flodin et al. 2025), i.e. in a seated position for the Q-NMES testing and lying in a prone position for the H- and G-NMES.

NMES was administered using a frequency of 36 Hz with a total stimulation time of eight seconds, including one second each for ramp-up and ramp down, with a pulse duration of 800µs (phase duration 400µs) and 18 s of rest time during the intervention. The NMES-level (0-999) represented a non-linear scale of current ranging from 0 to 120 milliampere (mA), provided by the manufacturer DJO Global.

### Intervention

The intervention was performed following identical procedure, time and using the same participants as described in our previous paper (Flodin et al. 2025). In brief, the order of testing for the three muscle groups (Q, H and G) was randomized, and NMES was applied to one of the muscle groups at a time, but simultaneously to both legs. Prior to S1, a pre-testing to determine the level of NMES intensity equal to a NMES-level below VAS 4 was determined on the specific muscle group for each participant. A warm-up session for two minutes (1 Hz, 15 mA) was performed, and then S1 and S2 were performed. The first 10 min of NMES treatment were administered at the intensity determined during pre-testing, and after 10 min, the participants were asked to re-rate their discomfort according to VAS. If the discomfort was lower, the NMES-intensity was increased to correspond to a level just below VAS 4. The intensity tolerated during the second 10 min compared to the first 10 min of each session on each of the muscle groups, were significantly higher as presented in our previous paper (Flodin et al. 2025). Mean intensities during the quadriceps, hamstrings, and gluteus muscle stimulation were 16.5, 20.5, and 25.4 mA, respectively. The level used was enough to elicit a visible muscle contraction for all participants. No significant correlations were found between the tolerated NMES-intensity and the outcome variables.

#### Time of the day for intervention

The intervention was started at different timepoint during the day for different participants. The earliest start time was 8.00 CEST (Central European Summer Time) and the latest was 17.00 CEST. For analysis, we divided the participants into two groups, those starting the intervention before 13.00 CEST (18 participants) and those starting after 13.00 CEST (18 participants) to investigate whether the start time of the day affected the outcome.

### Blood sampling

Before, and directly after the end of the NMES-session blood samples were collected from each participant. Before collecting the first sample, the participants remained seated and resting for 30 min. Two serum gel test tubes (8.5 ml, plastic, BD Vacutainer) were collected and inverted five times directly after collection at both time points.

### Analysis of blood samples

#### Serum samples

The collected blood samples were processed immediately after collection and 45 min clotting time by centrifugation at 2500 rpm (≈ 1250 g) for 10 min. After centrifugation, the plasma was separated, aliquoted into 500 µl vials, and then stored in a -80 C freezer.

#### Venous blood gas

One venous blood gas syringe (Line Draw Plus, 3 ml Luer-syringe with filter-pro), was collected and analyzed within 5 min after collection in a validated blood gas analyzer (ABL800 Flex, Radiometer Medical Denmark) (Neves et al. 2018). The sodium (mmol/l), potassium (mmol/l), lactate (mmol/l), pH and glucose (mmol/l) concentrations were measured.

#### OLINK Proteomics

The proteomic analyses were performed using the OLINK Proteomics multiplex inflammation panel which analyze 92 biomarkers using immunoassay where antibodies bind to their specific targets and can then be measured. In addition, Myoglobin that was analyzed in the Cardiovascular III panel as presented in our previous study (Flodin et al. 2025), was specifically assessed as well. The analyses were performed blinded at the Clinical Biomarkers Facility, Science for Life Laboratory, Uppsala University, with all analyses performed in the same session and in line with the description in a previous study (Hijazi et al. 2020). Protein levels were expressed as Normalized Protein expression (NPX). This method has been shown to have high reproducibility and repeatability, and good correspondence with conventional immunoassays (Assarsson et al. 2014; Siegbahn et al. 2017).

### Glucose metabolism related proteins

To further investigate the relationship between glucose metabolism and proteins related to inflammation and muscle damage, glucose metabolism related proteins was used. This since glucose itself, not being a protein, is not included in STRING database which only contains proteins. Glucose Transporters, Solute Carrier Family 2 Member 1–4 (SLC2A1, SLC2A2, SLC2A3, SLC2A4), Insulin (INS), Insulin receptor (INSR), Glycogen synthase GYS1 and GYS2 as well as proteins connected to glucose sensing and regulation Glucokinase (GCK), Glucokinase regulatory protein (GCKR) and marker of glucose metabolism Peroxisome proliferator-activated receptor gamma coactivator 1-alpha (PPARGC1A) were included.

### Statistics

Data was analyzed using SPSS version 27 (IBM Corp. Released 2016. IBM SPSS Statistics for Windows, Armonk, NY: IBM Corp.) and R 4.5.1. All variables were checked for skewness using the Shapiro-Wilks test. Normally distributed data were expressed as mean and standard deviation (SD) and p-values were calculated with paired t-test. Non-normally distributed data were presented using median (interquartile range), and p-values calculated with Wilcoxon signed test (paired). The significance level was adjusted with Bonferroni correction (0.05/92 = 0.000543 = < 5*10^4^) due to multiple comparisons. STRING v11.0 (https://string-db.org) was used for interaction analyses of significantly regulated proteins.

## Results

### Proteomics of inflammatory markers regulated by NMES

**Fig. 2:**
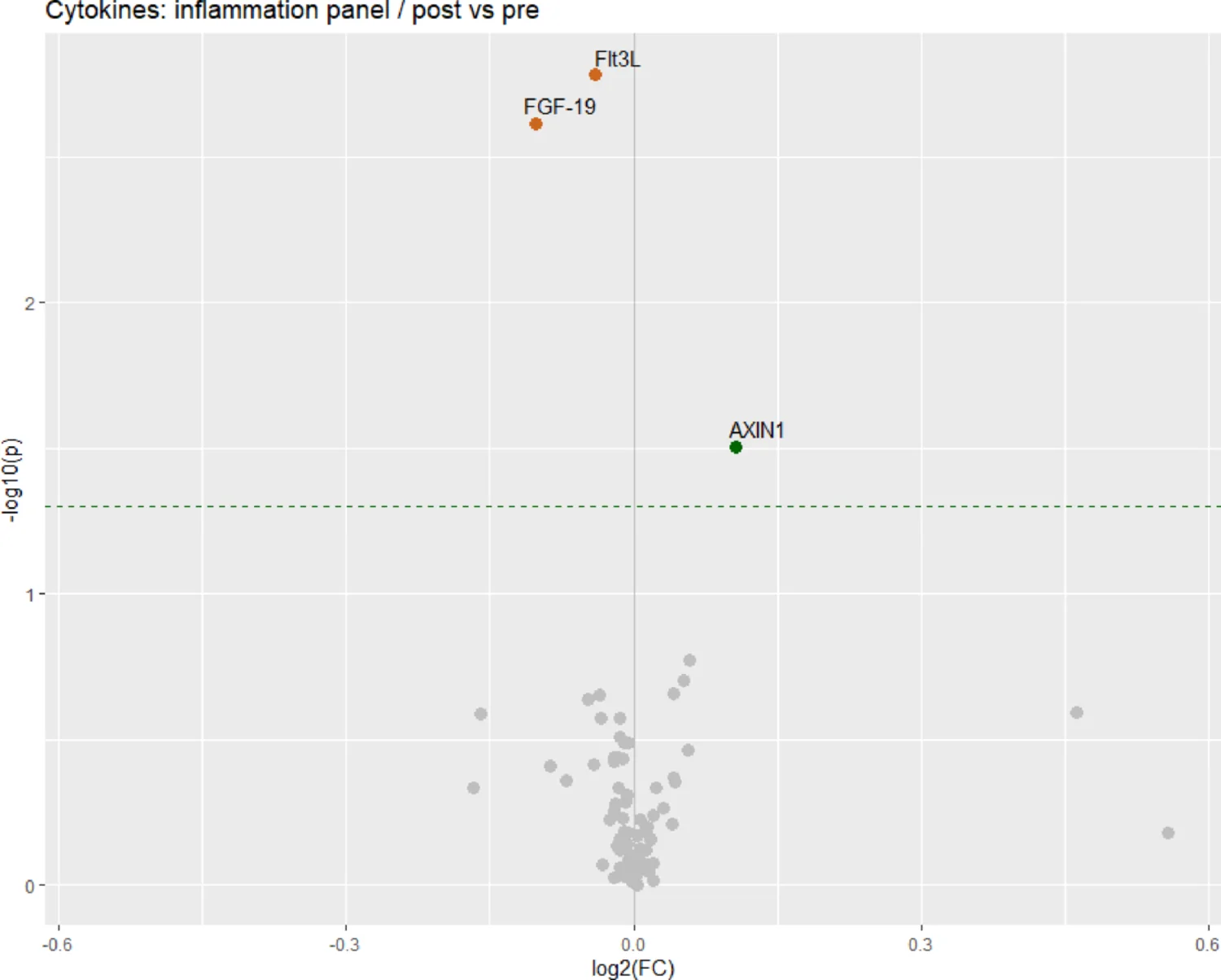
Volcano plot including the Olink proteomics inflammation panel differentially expressed by the neuromuscular electrical stimulation. *Flt3L* FMS-like tyrosine kinase 3 ligand, *FGF19* Fibroblast Growth Factor 19, *AXIN1* Axis Inhibition Protein 1

To investigate the effects of the 2-hour NMES-session on inflammatory markers, plasma proteins related to inflammation were analyzed using Olink proteomic analyses. Out of the 92 proteins included in the panel, 13 were differentially expressed by NMES treatment, with three proteins up- and ten downregulated (*p* < 0.05) (Table 2; Fig. 2).

To account for multiple testing Bonferroni correction (*p*<4 × 10^− 4^) was applied, and only two proteins remained significantly downregulated by the NMES treatment: FMS-like tyrosine kinase 3 ligand (Flt3L) and Fibroblast Growth Factor 19 (FGF19) (Table 2).

Further examination of the two significantly regulated proteins in relation to participant characteristics revealed that Flt3L was univariately negatively correlated to age (*R*= – 0.339, *p* = 0.043) and showed a trend toward negative correlation with BMI (*R*= – 0.300, *p* = 0.075). A linear regression model confirmed that higher age was associated with lower Flt3L values (R^2^ = 0.17, *p* = 0.013). No significant associations were observed for FGF19. No significant correlations were found between the time of blood sampling during the day and the protein levels.

**Table 2:** Differentially expressed proteins in the Olink proteomics inflammatory panel by the neuromuscular electrical stimulation

	Before NMES	After NMES	*p* value	Log2 fold change
Flt3L^a^	9.45 (9.52–9.75)	9.11 (9.01–9.34)	**5.6 × 10** ^**− 6**^	− 0.26
FGF19	9.43 (0.88)	8.80 (0.84)	**1.1 × 10** ^**− 5**^	− 0.64
FGF23^a^	0.90 (0.71–1.05)	0.82 (0.71–0.88)	0.003	− 0.09
CCL25^a^	7.09 (6.85–7.42)	7.08 (6.67–7.38)	0.004	− 0.16
S100A12	5.34 (0.64)	5.45 (0.66)	0.005	0.20
AXIN1	3.11 (0.41)	3.36 (0.55)	0.005	0.25
MMP10	9.12 (0.50)	9.01 (0.45)	0.008	− 0.10
CST5^a^	5.86 (5.55–6.15)	5.78 (5.49–6.05)	0.010	− 0.08
IL7	4.27 (0.43)	4.14 (0.48)	0.014	− 0.13
MCP1	13.0 (0.48)	12.9 (0.53)	0.015	− 0.13
LIF^a^	0.30 (0.18–0.45)	0.46 (0.33–0.60)	0.015	0.11
CXCL8^a^	5.57 (5.30–5.90)	5.50 (5.17–5.82)	0.034	− 0.14
TNFB	5.55 (0.39)	5.47 (0.37)	0.042	− 0.08

Data of protein value before and after NMES expressed as Normalized Protein expression (NPX)

*Flt3L* Fms-like tyrosine kinase 3 ligand, *FGF19* Fibroblast Growth Factor 19, *FGF23* Fibroblast Growth Factor 23, *CCL25* C-C Motif Chemokine Ligand 25, *S100A12* S100 Calcium Binding Protein A12, *AXIN1* Axis Inhibition Protein 1, *MMP10* Matrix Metallopeptidase 10, *CST5* Cystatin D (Cystatin 5), *IL7* Interleukin 7, *MCP1* Monocyte Chemoattractant Protein 1, *LIF* Leukemia Inhibitory Factor, *CXCL8* C-X-C Motif Chemokine Ligand 8, *TNFB* Tumor Necrosis Factor Beta

^a^Data expressed as Median (interquartile range). and p values calculated with Wilcoxon signed test (paired). The rest of the data expressed as mean (SD) and p-values calculated with paired t-test. Due to multiple comparisons the significance level was adjusted with Bonferroni correction (0.05/92 = 0.000543 = < 5 × 10^− 4^)

### Interaction between the inflammatory proteins differentially expressed by NMES

**Fig. 3:**
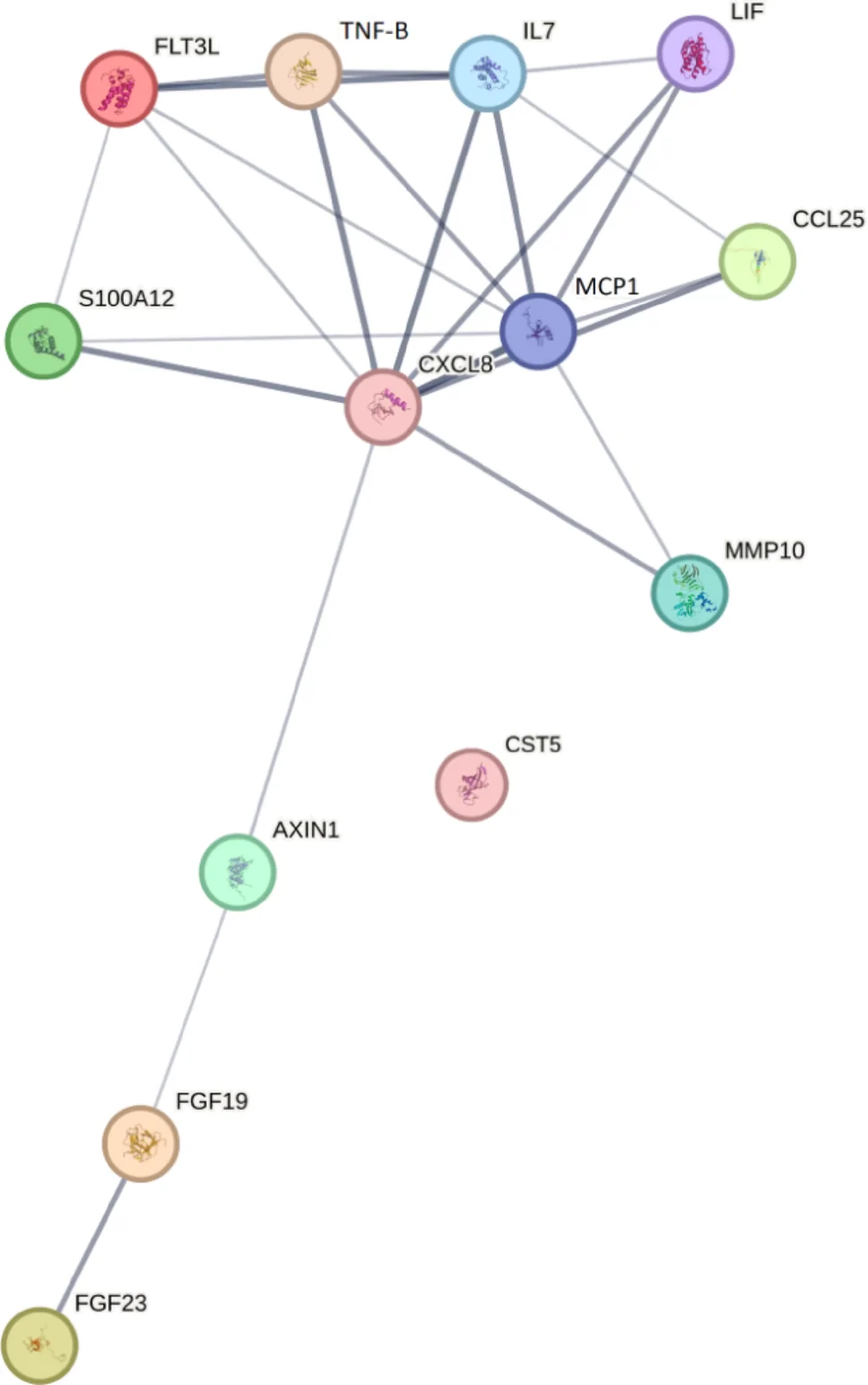
Protein-protein interaction network of the 13 differentially expressed proteins in Olink inflammation panel. Created in the STRING database. The line thickness indicates the strength of data support for the interaction. *Flt3L* Fms-like tyrosine kinase 3 ligand, *FGF19* Fibroblast Growth Factor 19, *FGF23* Fibroblast Growth Factor 23, *CCL25* C-C Motif Chemokine Ligand 25, *S100A12* S100 Calcium Binding Protein A12, *AXIN1* Axis Inhibition Protein 1, *MMP10* Matrix Metallopeptidase 10, *CST5* Cystatin D (Cystatin 5), *IL7* Interleukin 7, *MCP1* Monocyte Chemoattractant Protein 1, *LIF* Leukemia Inhibitory Factor, *CXCL8* C-X-C Motif Chemokine Ligand 8, *TNFB* Tumor Necrosis Factor Beta

To further explore the relationship between the inflammatory proteins differentially expressed by the NMES treatment, a protein-protein interaction (PPI) network was generated using the STRING database (Fig. 3). The analysis revealed, as expected, a complex network of associations among 12 of the 13 proteins. This was done in order to investigate potential interactions between the different proteins included in this study. The degree of connectivity within the PPI network varied among the identified proteins. For instance, the C-X-C Motif Chemokine Ligand 8 (CXCL8), also known as interleukin 8 (IL8), and Monocyte Chemoattractant Protein 1 (MCP1), also known as C-C Motif Chemokine Ligand 2 (CCL2), demonstrated extensive interactions with nearly all associated proteins, whereas FGF19 and FGF23 only connected with Axis Inhibition Protein 1 (AXIN1). Notably, the two proteins significantly regulated by NMES (FGF19 and Flt3L) demonstrated an indirect connection via CXCL8.

### Metabolic markers after NMES

To further assess the physiological metabolic response to NMES, additional blood analyses were performed for myoglobin, pH, lactate and electrolytes. Myoglobin levels increased significantly after NMES, reaching concentrations comparable to those typically observed after moderate exercise (*p* = 2 × 10^− 5^, Table 3) (Flodin et al. 2025). Electrolytes (sodium and potassium), blood pH and lactate remained unchanged. A modest but statistically significant decrease in blood glucose was observed following NMES (*p* = 0.039).

**Table 3:** Levels of metabolic markers

	Before	After	*p*value
Sodium	139.7 (1.92)	140.3 (0.21)	0.052
Potassium*	3.79 (3.65–4.05)	3.91 (3.75–4.03)	0.163
Lactate*	1.0 (0.7–1.1)	1.0 (0.8–1.2)	0.918
pH*	7.37 (7.35–7.40)	7.37 (7.35–7.38)	0.365
Glucose	5.35 (0.73)	5.17 (0.36)	**0.039**
Myoglobin (Flodin et al. 2025)	7.70 (0.50)	8.24 (0.83)	**2 × 10** ^**− 5**^

*Data expressed as Median (interquartile range). P values calculated with wilcoxon signed test (paired). The rest of the data expressed as mean (SD) and p values calculated with paired t test. The results from myoglobin were also presented in our previous article (Flodin et al. 2025)

### Glucose metabolism-inflammation interactions after NMES

**Fig. 4:**
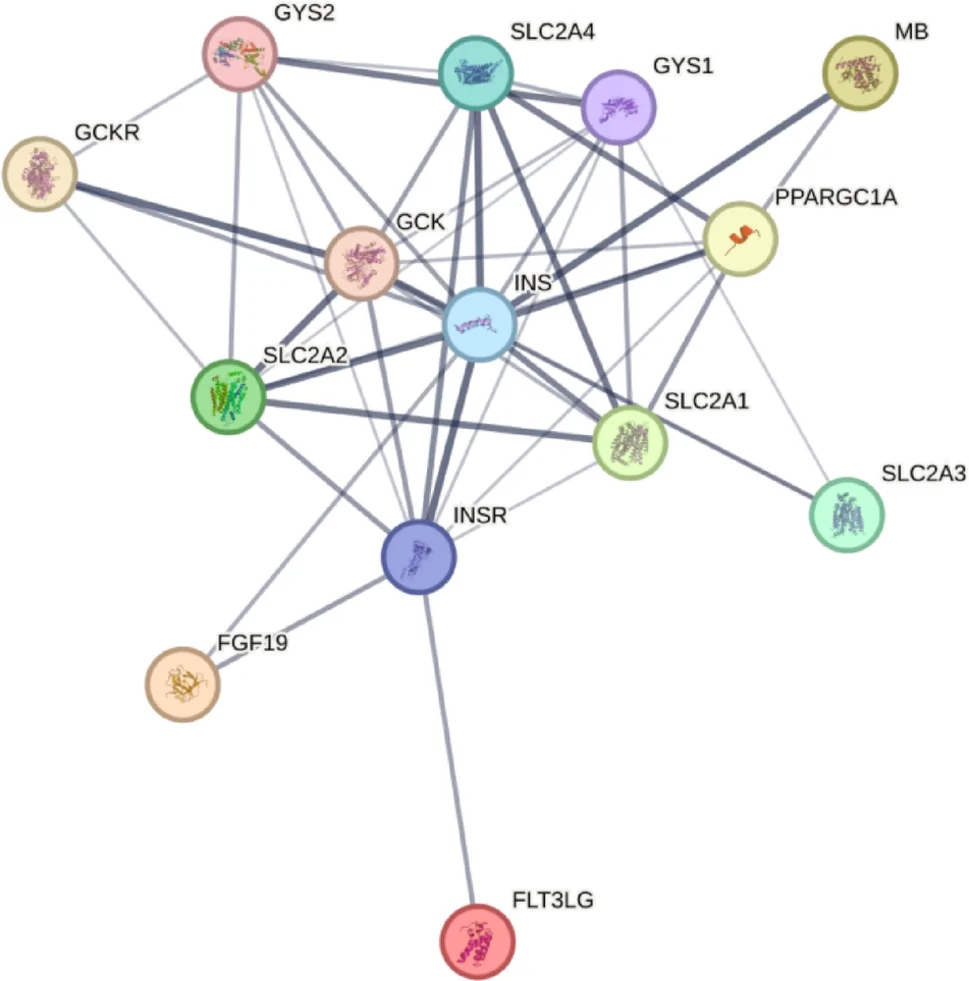
Protein-protein interaction network of the significantly regulated proteins from OLINK and glucose related proteins. Created in the STRING database. The line thickness indicates the strength of data support for the interaction. *Flt3L* FMS-like tyrosine kinase 3 ligand, *FGF19* Fibroblast Growth Factor 19, *MB* Myoglobin, *SLC2A1* Solute Carrier Family 2 Member 1, *SLC2A2* Solute Carrier Family 2 Member 2, *SLC2A3* Solute Carrier Family 2 Member 3, *SLC2A4* Solute Carrier Family 2 Member 4, *INS* Insulin, *INSR* Insulin Receptor, *GYS1* Glycogen Synthase 1, *GYS2* Glycogen Synthase 2, *GCK* Glucokinase, *GCKR* Glucokinase Regulatory Protein, *PPARGC1A* Peroxisome Proliferator-Activated Receptor Gamma Coactivator 1-Alpha

To further investigate potential relationships between glucose metabolism and the inflammatory markers regulated by NMES, a PPI-network was generated using the STRING database, including glucose-related proteins (Fig. 4). All significantly regulated markers - FGF19, Flt3l and myoglobin - showed associations with glucose metabolism. Both FGF19 and Flt3l were linked to INSR, while FGF19 and myoglobin interacted with insulin. Myoglobin was additionally connected to PPARGC1A, a key regulator of insulin action and cellular energy metabolism.

## Discussion

This study demonstrates that a single 2-hour session of submaximal NMES, delivered via wearable NMES-pants previously demonstrated to be well tolerated (Flodin et al. 2025), can modulate systemic inflammatory and metabolic pathways in healthy adults. Targeted proteomic profiling revealed significant downregulation of Flt3L and FGF19, accompanied by a modest increase in circulating myoglobin and a reduction in blood glucose. These findings indicate that NMES evokes systemic responses resembling those typically induced by physical exercise, both in terms of inflammatory and metabolic regulation (Ascensão et al. 2008; Del Coso et al. 2012; Yeung et al. 2002), likely involving glucose metabolism (Steensberg et al. 2003; Alharbi et al. 2023; Hamada et al. 2003).

The main finding of this study, using proteomic analysis, was the identification of 13 out of 92 inflammatory proteins in peripheral blood altered, with the majority being downregulated (10/13), by NMES. This overall dampening of inflammatory signaling is consistent with a stress-induced modulation, metabolic regulation or an inflammatory resolution phase rather than activation. Similar to exercise, previous transcriptomic data support that NMES induces an acute molecular response (Sillen et al. 2013; Flodin et al. 2024; Latimer et al. 2019; Karlsen et al. 2020). After adjustment for multiple comparisons, only Flt3L and FGF19 remained significant, both of which play key roles in metabolic regulation, suggesting that NMES may target systemic metabolic pathways.

Flt3L is involved in metabolism by regulating cell growth and differentiation, particularly in hematopoietic progenitor cells and dendritic cells, by increasing glucose uptake and glycolysis (McKenna et al. 2000). The finding of Flt3L downregulation after NMES may reflect an anti-inflammatory or recovery-related phase. To the best of our knowledge, no previous studies have investigated Flt3L in response to NMES. However, in studies investigating regular exercise, downregulation of Flt3L following exercise has been demonstrated in one previous study testing handgrip exercise (Landman et al. 2022), whereas other studies have reported upregulation of Flt3L after a single bout of whole-body exercise (Zaldivar et al. 2007; Bonsignore et al. 2002; Deckx et al. 2015). The discrepancy may be explained by the differences in type and extent of exercise, local handgrip exercise compared to whole-body exercise. In this context, NMES may be considered more analogous to localized exercise modalities.

The observed inverse correlation of Flt3L with age (*R* = -0.339, *p* = 0.043) and the trend with BMI (*R* = -0.300, *p* = 0.075) aligns with findings of age-related decline in metabolism (Beerman et al. 2010), and further suggest that participant characteristics modulate NMES responsiveness. Individualized NMES intensities may thus be needed to optimize metabolic effects across age and BMI ranges.

FGF19, a hepatokine central to glucose and lipid metabolism, has recognized antidiabetic and anti-obesity properties (Potthoff et al. 2011). The concurrent downregulation of FGF19 and mild reduction of blood glucose (*p* = 0.039), observed under fasting condition, may indicate increased peripheral glucose utilization during NMES. This interpretation aligns with reports that NMES enhances insulin sensitivity and glucose uptake (Sanchez et al. 2023), likely mediated by electrically stimulated muscle contractions, and mirrors responses seen during conventional exercise (Alharbi et al. 2023; Sanchez et al. 2023).

The finding of mild increase in myoglobin levels after NMES further strengthens the concept of an exercise-like effect of NMES. Myoglobin facilitates oxygen and fatty acid transport and supports mitochondrial respiration and ATP generation (Yamada et al. 2016; Wittenberg and Wittenberg 2003). The fact that NMES at a submaximal level induced only a mild increase in myoglobin, a marker of muscle activation/damage (Ascensão et al. 2008), without changes in systemic electrolytes, pH, or lactate, suggest that the NMES protocol was well-tolerated and without clinically relevant muscle damage, consistent with earlier findings (Flodin et al. 2024). The finding of non-elevated lactate levels may be related to the submaximal exercise level, but may also be related to other factors such as the resting intervals included in the protocol. Moreover, the physiological response seen resembles those observed after regular exercise (Ascensão et al. 2008; Del Coso et al. 2012). This indicates comparable mechanisms between exercise and NMES (Flodin et al. 2024) and also strengthens the conception that NMES, at least in healthy people, can be used without harm.

STRING-based PPI-analysis further revealed potential associations between FGF19, Flt3L and myoglobin with key glucose metabolism regulators, including insulin, INSR, and PPARGC1A, supporting a role for NMES in modulating metabolic signaling. Broader PPI analysis revealed a tightly interconnected inflammatory network among the differentially expressed proteins, with central roles played by chemokines such as CXCL8 (IL-8) and MCP-1 (CCL2). These proteins are established mediators of leukocyte trafficking and are commonly elevated in both acute and chronic inflammatory conditions (Zlotnik and Yoshie 2012). Post-NMES these two factors were downregulated, suggesting a mild anti-inflammatory shift. Although these changes did not remain significant after multiple analysis correction, the pattern supports a transition toward an inflammation-resolving state. Flt3L and FGF19 did not exhibit any direct interactions, but were linked via CXCL8, suggesting that NMES may exert its effects through intermediary chemokine signaling. This conclusion, however, warrants further studies.

Previous work using the same NMES set-up reported upregulation of Platelet endothelial cell adhesion molecule 1 (PECAM-1), Junctional adhesion molecule A (JAM-A), and Tissue inhibitor of matrix metalloproteinase 4 (TIMP4), together with downregulation of CCL15 and MMP3, indicating anti-inflammatory vascular responses (Laukoetter et al. 2007; Vetrano and Danese 2009; Shimizu and Dobashi 2012; Privratsky et al. 2010; Si-Tayeb et al. 2006; Eguchi et al. 2008; Flodin et al. 2025). Collectively, these findings reinforce the concept that NMES elicits transient inflammatory modulation followed by compensatory anti-inflammatory regulation - analogous to the adaptive pattern seen after exercise (Gliemann et al. 2018).

Although the study demonstrates clear molecular effects of NMES on the thigh and gluteus muscle, it is limited by the absence of a control group. It is also possible that the molecular effects may vary between other muscle groups (Borzykh et al. 2025), which was not examined in this study. In addition, the study included healthy participants aged between 18 and 60, which may not fully represent clinical populations. Although sampling was standardized, circadian variation cannot be fully excluded. We also only included one blood sampling timepoint after the invention in this study, and it is possible that the protein levels could vary over time after the intervention. Nevertheless, this study was the first to investigate the effects of NMES on these inflammatory markers and provides valuable insights into the effects of submaximal NMES regarding these factors. Future studies are needed to explore long-term effects, responses in clinical populations, and potential dose-response relationships tied to NMES intensity and duration.

## Conclusion

A single submaximal NMES session acutely modulated inflammatory and metabolic markers in a pattern consistent with exercise-induced adaptation. The significant downregulation of Flt3L and FGF19, along with decreased blood glucose and mild myoglobin elevation, indicate a systemic, metabolically active yet well-tolerated response to NMES. These findings support NMES as a safe, exercise-mimicking intervention with potential to mitigate inflammation and metabolic dysfunction in individuals unable to perform regular physical activity. Future studies should confirm these findings in larger and patient-based cohorts and define optimal NMES dose-response parameters.

## Abbreviations

AXIN 1: Axis inhibition protein 1
BMI: Body mass index
CCL: (2, – 25)-C-C Motif chemokine ligand (–2, – 25)
CEST: Central European summer time
CST5: Cystatin D (Cystatin 5)
CXCL8: C-X-C Motif Chemokine Ligand 8
FGF: (19, – 23)-Fibroblast Growth Factor (-19,-23)
FLt3L: FMS-like tyrosine kinase 3 ligand
GCK: Glucokinase
GCKR: Glucokinase regulatory protein
G: Gluteus
G: NMES-Neuromuscular electrical stimulation on the Gluteus muscle
GYS: (1, – 2)-Glycogen synthase (– 1,– 2)
H: Hamstrings
H: NMES-Neuromuscular electrical stimulation on the Hamstrings muscle
INS: Insulin
INSR: Insulin receptor
IL: (7, – 8)-Interleukin (–7, – 8)
JAM: A-Junctional adhesion molecule A
LIF: Leukemia inhibitory factor
MMP10: Matrix Metallopeptidase 10
MCP1: Monocyte chemoattractant Protein 1
MB: Myoglobin
NMES: Neuromuscular electrical stimulation
NPX: Normalized protein expression
PAS: Physical activity scale
PECAM: 1-Platelet endothelial cell adhesion molecule 1
PPARGC1A: Peroxisome proliferator-activated receptor gamma coactivator 1-alpha
PPI: Protein-protein interaction
Q: Quadriceps
Q: NMES-Neuromuscular electrical stimulation on the quadriceps muscle
S100A12: S100 Calcium binding protein A12
SLC2A: (1, – 2, – 3, – 4)-Solute Carrier Family 2 Member (– 1, – 2, – 3, – 4)
SD: Standard deviation
TIMP4: Tissue inhibitor of matrix metalloproteinase 4
TNFB: Tumor necrosis Factor Beta
VAS: Visual analogue scale
